# A Case of Delayed Diagnosis of Idiopathic Infantile Hypercalcemia Due to CYP24A1 Mutation: A 10-Year Journey

**DOI:** 10.7759/cureus.42811

**Published:** 2023-08-01

**Authors:** Zahid Khan, Gideon Mlawa, Yu-Hsuen Yang, Bashir Mahamud

**Affiliations:** 1 Acute Medicine, Mid and South Essex NHS Foundation Trust, Southend-on-Sea, GBR; 2 Cardiology, Barts Heart Centre, London, GBR; 3 Cardiology and General Medicine, Barking, Havering and Redbridge University Hospitals NHS Trust, London, GBR; 4 Cardiology, Royal Free Hospital, London, GBR; 5 Internal Medicine and Diabetes and Endocrinology, Barking, Havering and Redbridge University Hospitals NHS Trust, London, GBR; 6 Acute and General Medicine, Barking, Havering and Redbridge University Hospitals NHS Trust, London, GBR

**Keywords:** tc99m sestamibi scan, pet ct scan, hyperparathyroidism treatment, congenital hypercalcemia, cincalcet and pamidronate, renal and ureteral calculi, nephrocalcinosis (nc), cyp24a1 gene mutation, hypercalciuria, hypercalcemia

## Abstract

The parathyroid gland is responsible for the synthesis and secretion of parathyroid hormone, which is synthesized and released at an inverse relationship to the level of ionized calcium in the blood. Primary hyperparathyroidism affects women more than men. There are various causes for hyperparathyroidism-induced hypercalcemia and the most common cause is parathyroid adenoma. A less common cause of vitamin D-mediated parathyroid hormone-independent hypercalcemia is the loss of function mutation of the *CYP24A1* gene. The *CYP24A1* gene encodes the vitamin D 24-hydroxylase enzyme, responsible for hydroxylating the active form of vitamin D into an inactive form, and mutations in the *CY24A1* gene can lead to elevated active vitamin D metabolite levels. It can result in hypercalcemia and hypercalciuria-related complications. We present a case of a 72-year-old male patient referred to the endocrine clinic, who had repeated treatments for hypercalcemia and recurrent renal calculi. He underwent ultrasound, computerized tomography, and sestamibi scans, all reported as normal. Following this, the patient underwent a positron emission tomography (PET) scan, which was also normal. He then finally underwent genetic testing and tested positive for the *CYP24A1* gene. He was started on fluconazole 50mg once a day and cinacalcet 30mg twice with normalization of calcium level. Three of his family members also tested positive for the condition.

## Introduction

The causes of hypercalcemia include parathyroid adenoma, malignancy, and drugs. Infantile hypercalcemia was first described by Fanconi et al. and Lightwood in the early 1950s [1,2]. Patients classically present within the first year of life with profuse vomiting, irritability, constipation, poor weight gain, and nephrocalcinosis [3,4]. Although rare, it is an important cause of failure to thrive and can be lethal during the acute phase of hypercalcemia [5]. The exact prevalence and incidence of the condition remain unknown, although these are estimated to be very low, with 200 cases occurring over two years in the United Kingdom during the 1950s [6]. The introduction of routine vitamin D supplementation in fortified milk and other infant products during this period led to a significant increase in the number of cases. This phenomenon was reversed when the dose of Vitamin D supplementation was reduced on advice from the British Pediatric Association. It was, therefore, suggested that the condition might result from hypersensitivity to Vitamin D, although it was unclear whether its pathogenesis was due to excess activation or deficient inactivation of Vitamin D [6].

A distinction was subsequently made between cases of infantile hypercalcemia due to Williams-Beuren syndrome and Lightwood type, or idiopathic infantile hypercalcemia (IIH). The former is a syndrome that manifests a complex phenotype consisting of characteristic facies and supravalvular aortic stenosis and is associated with an early and severe presentation of infantile hypercalcemia [7]. The latter, which is the focus of this case report, presents with milder symptoms [4]. The link between *CYP24A1 *mutations and IIH has been recently established [8]. Vitamin D is converted to its active form by 25-hydroxylase in the liver, followed by 1ɑ-hydroxylase in the kidney. This form, 1,25-dihydroxy vitamin-D3, is then inactivated by 24-hydroxylase, an enzyme encoded by the *CYP24A1* gene on chromosome 20 [9,10]. Inactivating mutations or deletions in *CYP24A1* results in higher circulating active vitamin D, which binds to the vitamin D receptor to upregulate serum calcium levels.

Pre-clinical studies have previously shown that *CYP24A1* knockout (-/-) mice were prone to severe hypercalcemia leading to perinatal death, and nephrocalcinosis when supplemented with Vitamin D [11]. Schlingmann and colleagues demonstrated that loss-of-function mutations in *CYP24A1*, inherited in an autosomal recessive manner, led to the development of IIH in a cohort of infants [8].

In this study, we report a male English patient who presented with resistant hypercalcemia and recurrent renal stones. Investigations over 10 years were unable to identify a cause for these symptoms. Genetic testing revealed *CYP24A1* mutations, which confirmed a diagnosis of IIH. The patient got successfully treated with a combination of fluconazole and cinacalcet.

## Case presentation

A 72-year-old male with a history of hypercalcemia recently moved house and registered locally with a new general practitioner (GP) who referred him to the endocrine clinic in view of his history and high calcium level on his routine blood tests. He had recurrent admission with severe hypercalcemia and recurrent renal stones requiring hospital admission and had been in and out of hospital with hypercalcemia, renal colics, and haematuria for the past 11 years. His blood test revealed detectable/normal parathyroid hormone (PTH) 2.3 pcmol/L (reference range: 1.6-6.9 pmol/L) and calcium level was 2.89 mmol/L (reference range: 2.1-2.6 mmol/L). He was previously seen by urologists at another hospital for recurrent urinary tract infections (UTIs). The patient also complained of microscopic haematuria and underwent recent cystoscopy, which was normal. Other relevant past medical history included high cholesterol, type 2 diabetes mellitus, osteoporosis, folic acid deficiency, and hypertension. Regular medications included metformin 1g twice a day, losartan 50mg once daily, allopurinol 300mg once at night, linagliptin 5mg once a day, folic acid 5mg once a day, atorvastatin 20mg once at night, alendronic acid 70mg once weekly. His observations were normal and clinical examination was unremarkable. He had repeat blood tests in the clinic which showed a mildly elevated calcium level of 2.72 mmol/L. Laboratory tests results are shown in Table 1.

**Table 1:** Laboratory values for patient over a few months' time HbA1c: glycated haemoglobin;

Blood Test	April 2022	July 2022	November 2022	Reference range
Haemoglobin	135	142	138	133 – 173 g/L
White cell count	11.7	12.9	12.1	3.8 - 11 10*9/L
Platelet	3244	355	356	150 - 400 10*9/L
Neutrophils	7.2	7.6	8.1	2-7.5 10*9/L
Vitamin B12	420	-	435	191 - 663 ng/L
Urea	8.4	9.0	9.5	2.5 - 7.8 mmol/L
Creatinine	145	162	106	59 - 104 umol/L
Albumin	50	46	48	35 - 50 g/L
Sodium	142	138	137	133 – 146 mmol/L
Potassium	4.6	5.1	5.2	3.6-5.2 mmol/L
Magnesium	0.85	0.89	0.94	0.85-1.10 mmol/L
Adjusted calcium	2.52	2.65	2.77	2.1 - 2.6mmol/L
Phosphate (mmol/l)	1.20	1.08	1.18	0.8 - 1.5 mmol/L
Parathyroid hormone level	1.1	-	1.08	1.6 - 6.9 pmol/L
Total vitamin D	52	55	-	>50 nmol/L
Serum folate	> 20	> 20	-	3.9 - 26.8 ug/L
HbA1c	53	38	39	< 42 mmol/mol
Free T4	15.7	-	16	12 - 22 pmol/L
Thyroid-stimulating hormone	2.70	-	2.65	0.45 - 4.12 mU/L

**Figure 1:**
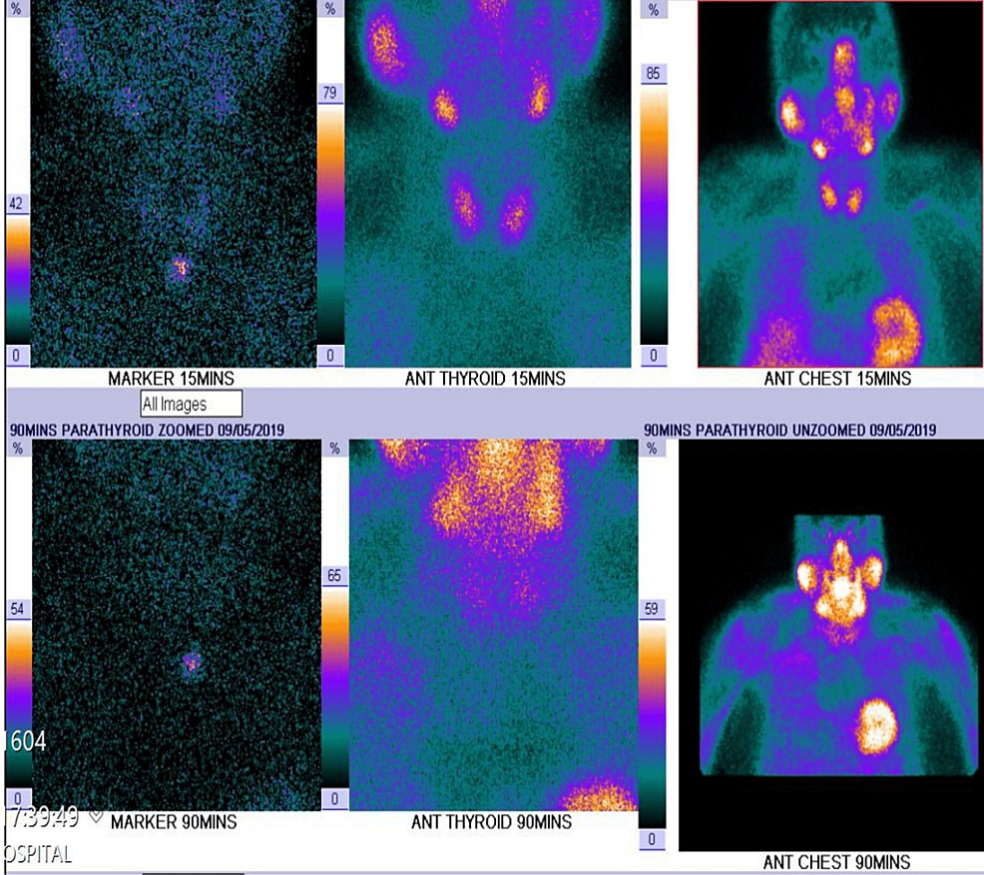
Technetium 99m sestamibi (MIBI) showing normal thyroid and parathyroid gland and normal technetium uptake

He had an ultrasound and technetium-99m sestamibi (MIBI) scan of the parathyroid gland which did not reveal any abnormality (Figure 1).

Computed tomographic (CT), chest abdomen pelvis (CAP), and positron emission tomography (PET) scans did not reveal any abnormality. He underwent genetic testing and was positive for *CYP24A1* mutation which reduces the activity of the vitamin D 24-hydroxylase enzyme, an enzyme responsible for vitamin D breakdown. The patient was diagnosed with infantile hypercalcemia, which is an autosomal recessive disorder. The patient was started on fluconazole 50mg once a day and cinacalcet 30mg twice with normalization of calcium level in a few weeks' time. His family members and first-degree relatives were offered genetic screening and three tested positive for the condition. His calcium level normalized following the initiation of the above treatment; the latest calcium level was 2.50 mmol/L, and he did not require any further admission for urinary calculi.

## Discussion

Patients with IIH demonstrate typical laboratory findings of raised serum calcium, suppressed PTH, mildly elevated levels of 1,25-dihydroxyvitamin D3, and hypercalciuria. IIH used to be a diagnosis of exclusion once more common causes of hypercalcemia were excluded but can now it be diagnosed using *CYP24A1 *genetic testing [11,12].

The natural history of IIH is poorly understood due to the condition’s rarity. A case series of 11 children from Australia suggests that hypercalcemia should resolve for most children by three years of age, although most individuals will continue to experience persistent hypercalciuria, nephrolithiasis, and nephrocalcinosis. Further, patients may experience mild-to-moderate intellectual disabilities with a reduced intellectual quotient compared to the general population [12].

The treatment of IIH in the acute hypercalcemia phase is per standard hypercalcemia management, which consists of aggressive hydration and loop diuretics. Should these measures fail, other options include the use of corticosteroids, calcitonin, bisphosphonates, imidazoles such as ketoconazole, triazoles such as fluconazole, and even hemodialysis [12]. The link between vitamin D and phosphate metabolism was illustrated by mutations in *CYP24A1* and *SLC34A1* affecting the production of 24-hydroxylase enzyme and sodium-dependent phosphate transporter 2A (NaPi-IIa) protein, which can cause autosomal recessive infantile hypercalcemia types 1 and 2 [13].

Imidazoles and triazoles are anti-fungal agents which also inhibit 1ɑ-hydroxylase and 25-hydroxylase involved in activating vitamin D and have been used to treat IIH with good effect [14-16]. These treatments, however, run the risk of causing adrenocortical insufficiency due to their inhibitory effect on endogenous corticosteroid synthesis and are also hepatotoxic when used in the longer term [17,18]. Calcitonin is an endogenous hormone produced by the parafollicular cells of the parathyroid gland and opposes the effect of PTH and 1,25-dihydroxy vitamin D3 to drive down calcium levels. The use of recombinant calcitonin has been reported to be effective in the management of IIH [18]. Long-term management involves placing patients on low-calcium and vitamin D-free diets. Patients with persistent hypercalciuria and recurrent nephrolithiasis can be treated with thiazide diuretics, which tend to reduce urinary calcium excretion [19].

Meusburger et al. published a case report of a 29-year-old patient who got investigated for raised creatinine level and previous history of failure to thrive, diabetes insipidus, hypertension, and hypercalcemia. He underwent *CYP24A1* gene testing and was found to be homozygous for three common DNA polymorphisms (rs2296241, rs2762934, and rs6022987) and an undescribed nonsynonymous mutation in exon 4, causing tryptophan replacement with arginine in codon 210 [20].

Molin et al. published a study including 72 patients with chronic hypercalcemia and investigated them for *CYP24A1* mutations. They concluded that patients with hypercalcemia, low PTH, and renal disease are more likely to have *CYP24A1* biallelic mutations. They noted *CYP24A1* variations in 25 (35%) patients with hypercalcemia, and 20 (28%) patients had biallelic variations, mainly patients with nephrocalcinosis or renal stones [21]. In another case report, a 33-year-old Egyptian patient with hypercalcemia, recurrent nephrolithiasis, nephrocalcinosis, and myocarditis was diagnosed with IIH, and two first-degree relatives identified with similar history [22]. These patients usually respond to fluconazole, and patients with marked severe hypercalcemia should receive a course of prednisolone to inhibit 1,25-(OH)2-vitamin D levels [22].

Fluconazole is primarily an antifungal agent belonging to the group azoles which are heterocyclic ring compounds. They are classified based on the number of nitrogen atoms in the azole rings to either imidazoles (e.g. ketoconazole) or triazoles (e.g. fluconazole), containing two or three nitrogen atoms, respectively [16]. They exhibit their antifungal action through the inhibition of lanosterol 14-alpha demethylase, a cytochrome P450 enzyme for the synthesis of a fungal plasma membrane constituent, which can then inhibit cytochrome 450-dependent enzyme systems that are involved in vitamin D hydroxylation. This led to using ketoconazole and fluconazole to manage hypercalcemia, especially in patients with hyperparathyroidism and sarcoidosis [16]. Ketoconazole is less commonly used due to serious side effects such as hepatotoxicity compared to fluconazole. 

The primary aim of cinacalcet in primary hyperparathyroidism (hyperparathyroidism) is to lower the level of PTH and calcium in the blood thus preventing or minimizing the symptoms of hypercalcemia and hyperparathyroidism [23]. Interestingly, cinacalcet does not reduce the risk of renal calculi in patients with hyperparathyroidism and endocrinologists need to pay attention to this separately. It is also important to be careful in prescribing cinacalcet to patients with recurrent renal calculi due to the increased risk of renal calculi because of increased urinary calcium excretion. Cinacalcet mimics the action of calcium and can activate the calcium receptors by binding to them on parathyroid cells thus suppressing the production of PTH. 

## Conclusions

Infantile hypercalcemia is a rare autosomal recessive disorder. Patients with chronic hypercalcemia, recurrent nephrocalcinosis, and renal calculi should be offered genetic screening for familial causes of hypercalcemia including infantile hypercalcemia. Most patients get diagnosed in early childhood; however, in rare cases, adults too can present with this condition. Genetic screening should be offered to first-degree relatives of these patients due to the autosomal recessive inheritance of the disorder.
